# Ingrowing Eye Lashes

**Published:** 1869-10

**Authors:** 


### INGROWING EYE LASHES.

Plucking out the eye lashes is a very prevalent habit among persons afflicted with "weak eyes," under the belief that they are "wild hairs" and the source of irritation to their eyes. Almost every neighborhood has its expert in the plucking-out-business, who, under the direction of some old granny, plies his vocation with marvelous skill, but almost certain destruction to the eyes. He imagines and declares that the lashes "*grow through the lids and scratch the eye-ball beneath.*" Nothing could be more absurd, and we desire to assure our readers that there is no such thing as "wild hairs"

of the eye lids, and that the less plucking out of lashes they engage in, the better it will be for their visual organs.

The habitual removal of the eye lashes distorts the margin of the lid, and changes the direction of the cilia or hairs, so that when they reappear—as they are sure to do—grow in upon the eye, acting as a constnat irritant, requiring their removal then, every two or three days, from necessity. We have seen hundreds of unfortunate creatures who have led a life of constant pain and annoyance, from the simple indiscretion of plucking out a few harmless eye lashes in early life, for the relief of some slight irritation of the eye, believing them to be "wild hairs."

The pricking of the eye, which causes persons to imagine the presence of hairs in contact with it, is usually caused by what is known as the *granular lid*—a roughened condition of the under surface of the lid, which irritates and inflames the eye; so that no possible benefit can be derived from the removal of the eye lashes; yet, when such a proceeding has been rendered necessary, from repeated interference with them a surgical operation can be performed, by means of which the lid can be restored to its natural shape, and the offending lashes elevated from their contact with the eye.

Persons troubled with such a deformity of the lids, should not pluck out the offending lashes, as they speedily reappear with their sharpness intensified, but should secure the services of any competent surgeon, and so relieve themselves of a world of agony, and at the same time preserve their vision, which would otherwise sooner or later be destroyed.

